# Quantitative plasma profiling by ^1^H NMR-based metabolomics: impact of sample treatment

**DOI:** 10.3389/fmolb.2023.1125582

**Published:** 2023-06-02

**Authors:** Francisco Madrid-Gambin, Sergio Oller, Santiago Marco, Óscar J. Pozo, Cristina Andres-Lacueva, Rafael Llorach

**Affiliations:** ^1^ Applied Metabolomics Research Group, IMIM—Institut Hospital del Mar d’Investigacions Mèdiques, Barcelona, Spain; ^2^ Signal and Information Processing for Sensing Systems, Institute for Bioengineering of Catalonia (IBEC), The Barcelona Institute of Science and Technology, Barcelona, Spain; ^3^ Department of Electronics and Biomedical Engineering, Faculty of Physics, University of Barcelona, Barcelona, Spain; ^4^ Biomarkers and Nutrimetabolomics Laboratory, Department of Nutrition, Food Science and Gastronomy, Faculty of Pharmacy and Food Sciences, Campus Torribera, University of Barcelona, Sant Coloma de Gramanet, Spain; ^5^ Food Innovation Network (XIA), Santa Coloma de Gramanet, Spain; ^6^ Institut de Recerca en Nutrició i Seguretat Alimentària (INSA-UB), Santa Coloma de Gramanet, Spain; ^7^ Centro de Investigación Biomédica en Red de Fragilidad y Envejecimiento Saludable (CIBERFES), Instituto de Salud Carlos III, Madrid, Spain

**Keywords:** metabolomics, nuclear magnetic resonance, plasma, quantitative analysis, quantification, sample treatment

## Abstract

**Introduction:** There is evidence that sample treatment of blood-based biosamples may affect integral signals in nuclear magnetic resonance-based metabolomics. The presence of macromolecules in plasma/serum samples makes investigating low-molecular-weight metabolites challenging. It is particularly relevant in the targeted approach, in which absolute concentrations of selected metabolites are often quantified based on the area of integral signals. Since there are a few treatments of plasma/serum samples for quantitative analysis without a universally accepted method, this topic remains of interest for future research.

**Methods:** In this work, targeted metabolomic profiling of 43 metabolites was performed on pooled plasma to compare four methodologies consisting of Carr-Purcell-Meiboom-Gill (CPMG) editing, ultrafiltration, protein precipitation with methanol, and glycerophospholipid solid-phase extraction (g-SPE) for phospholipid removal; prior to NMR metabolomics analysis. The effect of the sample treatments on the metabolite concentrations was evaluated using a permutation test of multiclass and pairwise Fisher scores.

**Results:** Results showed that methanol precipitation and ultrafiltration had a higher number of metabolites with coefficient of variation (CV) values above 20%. G-SPE and CPMG editing demonstrated better precision for most of the metabolites analyzed. However, differential quantification performance between procedures were metabolite-dependent. For example, pairwise comparisons showed that methanol precipitation and CPMG editing were suitable for quantifying citrate, while g-SPE showed better results for 2-hydroxybutyrate and tryptophan.

**Discussion:** There are alterations in the absolute concentration of various metabolites that are dependent on the procedure. Considering these alterations is essential before proceeding with the quantification of treatment-sensitive metabolites in biological samples for improving biomarker discovery and biological interpretations. The study demonstrated that g-SPE and CPMG editing are effective methods for removing proteins and phospholipids from plasma samples for quantitative NMR analysis of metabolites. However, careful consideration should be given to the specific metabolites of interest and their susceptibility to the sample treatment procedures. These findings contribute to the development of optimized sample preparation protocols for metabolomics studies using NMR spectroscopy.

## 1 Introduction

Metabolomics refers to the investigation of low-molecular-weight compounds present in biological samples (Wishart, 2019). Liquid chromatography-tandem mass spectrometry (LC-MS) and nuclear magnetic resonance (NMR) are popular techniques to explore alterations in the metabolome (Guijas et al., 2018). Metabolomic studies applied to human research generally employ biological samples such as urine, blood, saliva, and feces, among others. Often, extraction of metabolites from different matrices, such as blood, is necessary (Vignoli et al., 2019).

Blood-based samples—plasma and serum—are key in human metabolomics investigations (Zhang et al., 2012). Blood is the primary carrier of small compounds in the human body. This matrix carries nutrients, hormones, dissolved gases, and breakdown products, and it is involved in regulating body temperature, pressure, pH stabilization, and defense system, among other functions (Martini and Ober, 2006). Plasma/serum components are dysregulated by the presence of dysfunctions in the organism and/or pathological conditions (Psychogios et al., 2011). This makes these biofluids appropriate samples for the study of mechanisms of action, disease characterization, and the discovery of biomarkers of a specific condition through comprehensive fingerprinting (Scalbert et al., 2014; Yan and Xu, 2018; Attard et al., 2019; Aderemi et al., 2021).

Plasma/serum samples are also rich in other nonpolar molecules such as cholesterol and triglycerides, that need to be transported in the biofluid associated with various lipoprotein particles. Lipoproteins have, in general, heavier molecular weight than polar metabolites. They are divided based on size, density, and their relative content of triglycerides, cholesterol, and protein into high-density lipoproteins (HDL), low-density lipoproteins (LDL), intermediate-density lipoproteins (IDL), very-low-density lipoproteins (VLDL), and chylomicrons (Cox and García-Palmieri, 1990). The plasma/serum lipid fraction is a subject of systematic study in another omics technology, lipidomics. The lipoprotein profile can be measured when lipids and lipid composition change as a result of pathological states or disorders (Li et al., 2017). One of the most recent and applicable examples is the COVID-19 disease (Bruzzone et al., 2020; Kimhofer et al., 2020; Meoni et al., 2021). However, the presence of these macromolecules implies a great challenge to exploring low-molecular-weight metabolites (Ulaszewska et al., 2019; Ulmer et al., 2021). For this latter purpose, prior extraction of the low-molecular-weight metabolites from this matrix is necessary to be able to detect and, if necessary, quantify such metabolites.

The International Organization for Standardization (ISO) has already published specifications for pre-examination processes for metabolomics in urine, venous blood serum, and plasma (ISO 23118, ISO, 2021); however, these recommendations are yet to be universally adopted. In a multicenter study performed by Ghini et al. (2022), they examined how different operating procedures, such as different collection tubes, processing time, and storage conditions, affected the level of metabolites in serum and plasma samples (Ghini et al., 2022). In addition to these differences in the stage of sample pre-processing, sample treatment also affects the final metabolite levels. The reliability of the sample treatment utilized for the extraction requires that the chemical nature and relative concentration of metabolites found in the plasma/serum samples stay unaltered by the technique. However, in NMR-based metabolomics, the consistent quantification of plasma/serum metabolites remains a difficult concern (Ghini et al., 2019; Gowda et al., 2021), finding differences among sample treatments (Nagana Gowda and Raftery, 2014). Proteins and phospholipids show a large number of mostly broad NMR peaks, which overlap with the resonances of low-molecular-weight metabolites. They introduce an unstable spectral baseline, which makes identification and quantification of these metabolites difficult, and in addition, proteins may interact with the peak intensity of specific metabolites (Tiziani et al., 2008; Nagana Gowda and Raftery, 2014).

One of the approaches addressed in NMR may be partially overcome by a spin-spin relaxation edition, using the Carr-Purcell-Meiboom-Gill (CPMG) pulse sequence (Wang et al., 2003). Nevertheless, this approach has important drawbacks, such as the intensity loss of peak signals, the waste of relevant information about the chemical nature of phenolic conjugates, and the introduction of artifacts. Several metabolites such as tyrosine, histidine, and lactate, may bind to plasma/serum proteins, and consequently, the absolute concentration of several metabolites is undervalued and unreliable without cleaving the bonds to proteins (Bell et al., 1988; Nicholson and Gartland, 1989; Chatham and Forder, 1999). Similarly, trimethylsilylpropanoic acid (TSP), the quantification standard commonly utilized in aqueous solutions such as plasma/serum, displays the tendency to bind to protein (Wallmeier et al., 2017). Hence, the utilization of TSP as quantification standard in plasma/serum samples acquired by the CPMG sequence presents several challenges. Firstly, chemical shift variations can occur in TSP when using CPMG sequences due to pulse imperfections and relaxation differences during refocusing periods. These variations may deviate from the expected chemical shift of TSP at around 0 ppm, potentially introducing errors in quantification. Moreover, elimination of macromolecules that are bound to TSP clearly causes changes in TSP intensity and peak shape. Such interference compromises the accuracy and reliability of quantification using TSP as an internal standard (Gowda et al., 2021). Suitable experimental designs and appropriate data processing techniques may be required to overcome these challenges and improve the reliability in CPMG sequences. Exploring alternative internal standards may enhance the accuracy of quantification. An option mostly preferred for quantitative NMR are maleic, fumaric and formic acids, but to date has been poorly utilized in metabolic studies (Bliziotis et al., 2020; Gowda et al., 2021). On the other hand diffusion-weighted NMR pulse sequence has been also used to detect signals from only the macromolecular components, e.g., lipoproteins (Liu et al., 1996), and along with computational algorithms, it may be useful in quantitative metabolomics (de Graaf and Behar, 2003). Nevertheless, diffusion sensitization easily erases signals from fast-diffusing metabolites and slow-diffusing lipoproteins, requiring distinct diffusion coefficients for each chemical structure (de Graaf et al., 2015) and has not yet been tested for metabolites at concentrations below 10 μM. Several sample treatments have emerged to remove proteins and phospholipids from serum/plasma.

A more comprehensive range of metabolites can be quantified by ultrafiltration compared to CPMG by removing macromolecules with filters (Psychogios et al., 2011). However, due to its time-consuming filtration step that requires expensive filters, this gold standard has several limitations, including laboriousness and cost (Wallmeier et al., 2017). Moreover, likewise as for CPMG editing, the application of ultrafiltration may underestimate certain metabolites bound to macromolecules. Alternatively, precipitation with organic solvents have been primarily applied for quantifying plasma/serum metabolites before NMR analysis (Nagana Gowda and Raftery, 2014; Nagana Gowda and Raftery, 2017). A less popular technique is the addition of electrically charged silica nanoparticles, which can be utilized combined with ultrafiltration and solvent precipitation for the aggregation and co-precipitation of proteins (Zhang et al., 2016). Although this treatment exhibited spectral improvements at the qualitative level, the technique performance does not allow quantitative analysis by itself.

In MS, glycerophospholipid-SPE (g-SPE) has been described, and compared to ultrafiltration and solvent extraction with different gradients, finding that SPE-mediated phospholipid removal was the treatment with the best coverage of non-lipid metabolites, extraction reproducibility, and minimization of matrix effects (Tulipani et al., 2013; González-Domínguez et al., 2020). Although the SPE method has led to considerable improvements in MS-based metabolomics (Liu et al., 2019), this methodology has not yet been described for treating plasma/serum before quantitative NMR analysis. Considering that sample treatments in quantitative NMR plasma analysis may involve metabolite-dependent particularities, this topic remains of interest, especially for targeting a particular set of metabolites (Nagana Gowda and Raftery, 2017). This work aimed to evaluate the performance of four procedures to remove plasma proteins and phospholipids, CPMG, ultrafiltration, protein precipitation, and g-SPE in pooled human plasma. To this end, each sample treatment combined with ^1^H-NMR analysis enabled the quantitation of up to 40 metabolites, including amino acids, carboxylic acids, and short-chain fatty acids, among other compounds.

## 2 Materials and methods

### 2.1 Subjects

Venous blood samples from five non-smoking fasting healthy volunteers with 37.9 ± 10.3 years of age (mean ± SD) were collected into heparin-containing vials at the Hospital Clinic of Barcelona (Spain). Exclusion criteria included serious illness, supplement intake, medication, and pregnancy. Plasma samples were acquired after the removal of cells by centrifugation at 1,600 *g* for 15 min at room temperature. Pooled plasma aliquots were used to avoid biological variability in the comparative analysis among sample preparation procedures, isolating variability derived from the techniques. Aliquots were stored at −80°C for analysis and processing.

The Bioethical Committee of the University of Barcelona approved the research protocol, and all the participants provided written informed consent. The study was inscribed as ISRCTN17200423 in the International Standard Randomized Controlled Trial Number registry.

### 2.2 Sample treatment

The different sample treatments of plasma samples were conducted independently.

*Protein precipitation with methanol*. Four 200 μL of pooled plasma were thawed, vortexed, mixed with methanol in a 2:1 solvent-to-plasma ratio (v/v), and incubated at −20°C for 20 min. The samples were centrifuged at 13,400 *g* for 30 min to pellet macromolecules and proteins. Supernatants were decanted into new vials and dried under a nitrogen stream. Dried samples were mixed with 100 μL of phosphate buffer in D_2_O, which contained 2.32 mM of 3-(trimethylsilyl)-proprionate-2,2,3,3-d_4_ (TSP), and the pH was adjusted to 7.0.

*Glycerophospholipid SPE*. SPE procedure using Ostro^®^ 96 plate (Ostro plates, Waters) for protein precipitation and phospholipid removal plates was adapted to NMR-based metabolomics spectroscopy based on a previously published procedure for LC-MS (Tulipani et al., 2013). Four 200 μL aliquots of pooled plasma were thawed, vortexed, and mixed with acidic solvent, followed by g-SPE with pressure valves. Samples were pipetted into the plate wells, followed by the forceful addition of 1% formic acid in acetonitrile 3:1 solvent-to-plasma ratio (v/v). After drying, samples were mixed with 100 μL of the phosphate buffer in D_2_O (pH 7.0) containing 2.32 mM of TSP.

*Ultrafiltration*. Ultrafiltration was applied based on a published methodology with modifications (Nagana Gowda and Raftery, 2014). Centrifugal filters (3 kDa cutoff; Amicon Microcon, YM-3; Sigma-Aldrich) were initially washed with distilled water. Additionally, filter tubes were centrifuged three times with 500 μL of distilled water, each time at 13,400 g for 20 min, to remove residual glycerol from filters. Four 200 μL of pooled plasma were then transferred to filter tubes and centrifuged as previously described. The filtrates were mixed with 100 μL of the phosphate buffer.

*Untreated*. Four 200 μL of pooled plasma were then transferred to filter tubes and centrifuged as previously described. The filtrates were mixed with 100 μL of the phosphate buffer, utilizing 0.3 μM of formic acid as internal standard.

All the solutions were individually made up to 600 μL with D_2_O and transferred to 5 mm NMR tubes.

### 2.3 NMR spectra acquisition

All NMR experiments were performed at 298 K on a Bruker Avance Neo 400 MHz spectrometer equipped with a cryoprobe. One-dimensional proton spectra were acquired using a pulse sequence (1D NOESY) with presaturation of the water resonance. Additionally, untreated plasma samples were acquired using a CPMG pulse sequence. For both sequences, an exponential window function was applied to the free induction decay (FID) with a line-broadening factor of 0.3 Hz prior to the Fourier transformation. For each spectrum, FIDs were collected into 32 K data points (128 scans) with a spectral width of 14 ppm, an acquisition time of 3 s, a relaxation delay of 5 s, and a mixing time of 10 ms. Parameters were adjusted to allow metabolite quantification by Chenomx NMR Suite 10 profiler (Chenomx Edmonton, Canada). All the NMR spectra were phased, baseline corrected, and calibrated (TSP, 0.0 ppm) using TopSpin software (version 3.6, Bruker, BioSpin, Germany).

### 2.4 NMR metabolic profiling and quantitation

Identification and quantitation of detected metabolites were performed using Chenomx NMR Suite 10 profiler. Chenomx is a robust, reliable, and widely spread software for NMR-based metabolite quantification (Jung et al., 2016; Crook and Powers, 2020). This software enables metabolite deconvolution in complex samples and can determine concentrations in overlapped spectral regions. The software fits the peaks with a set of model spectra characterizing the chemical environments of each metabolite. Finally, the software calculates the concentrations of each metabolite using the peak areas and the calibration curves generated from the model spectra.

The fit of spectral signals was performed with a standard metabolite library for 400 MHz ^1^H NMR spectra. For treated plasma samples, peak fitting with reference to the internal TSP signal allowed quantification of absolute concentrations for all identified metabolites. For untreated samples variations in previous formic acid were standardized (blank) and the formic acid signal (δ8.44, 35.05 Hz) was utilized for untreated plasma samples. Further contributions to the proton peak assignment were provided by comparing the chemical shifts with those available in the Human Metabolome Database (http://www.hmdb.ca).

### 2.5 Data analysis

The dataset was imported to R software version 4.2 for statistical analyses. The coefficient of variation (CV) was calculated to determine the replicability for each metabolite and those with a CV > 20% in more than one group were subsequently removed from the multiclass analysis. The differences in metabolite concentration associated with the different treatments were evaluated using the Fisher Score (Tang et al., 2014). In order to provide stable estimates of the statistical properties, permutation tests with random sampling with replacement. This method is often used when the sample size is compromised to increase the effective sample power (Dwivedi et al., 2017; Anderson and Verbeeck, 2023). We performed 5,000 multiclass permutations on Fisher Scores to distinguish their sensitivity to sample treatment. Original Fisher Scores were compared with Fisher Scores randomly distributed, and 95% interval confidence and *p*-values were determined from permutations. Pairwise assessments of significant metabolites were applied to test binary permutation test (5,000 binary permutations) of Fisher Scores. The Benjamini–Hochberg procedure was carried out on all analyses to control the false discovery rate (FDR) (Benjamini and Hochberg, 1995). An FDR-corrected *p*-value < 0.05 was considered statistically significant.

## 3 Result and discussion

**FIGURE 1:**
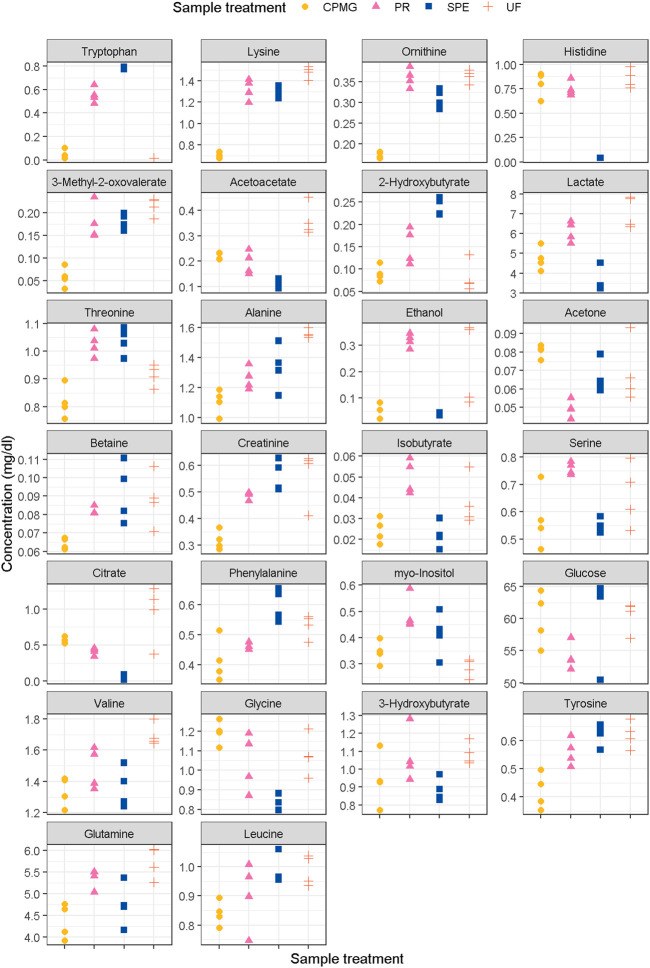
Concentrations of metabolites with a top-10 fisher score in any of the comparisons (extension in Supplemental Figure S1). PR, methanol precipitation; SPE, glycerophospholipid-solid phase extraction; UF, ultrafiltration.

This study focused on investigating the influence of different sample treatments on the concentrations of a panel of 43 metabolites found in human plasma (Supplemental Figure S1). Compounds, concentrations (mg/dL), and CV (%) are detailed in Table 1 and Figure 1. Based on the present results, methanol precipitation exhibited a number of six compounds with a CV > 20%, while ultrafiltration presented eight compounds with a CV above this threshold. The metabolites meeting this criteria in at least three of the methodologies corresponded with acetate, isobutyrate and N,N-dimethylglycine, and therefore, did not meet the benchmark for acceptable precision in analytical methods (Bioanalytical Method Validation Guidance for Industry FDA, 2023; European Medicine Agency, 2011; ICHH Guideline, 2019). Although acetate is a volatile short-chain fatty acid, it is generally considered stable for quantification under standard storage and handling conditions (Psychogios et al., 2011). However, acetate can be subject to degradation or changes in concentration over time if not properly handled or stored (Bernini et al., 2011). Factors such as temperature, pH, exposure to air or light, and the presence of enzymes or microorganisms can affect the stability of acetate (Brunius et al., 2017). As with acetate, isobutyrate, methanol, and compounds with low-molecular weight (<90 g/mol) such as 2-hydroxybutyrate and ethanol, and might be affected by the evaporation process of the sample. Remarkably, the average concentration of 2-hydroxybutyrate after ultrafiltration and CPMG was three times lower compared to g-SPE. The measure of this metabolite was uniquely precise in the g-SPE (0.240 ± 0.019 mg/dL, CV of 8.1%) and CPMG (0.090 ± 0.017 mg/dL, CV of 19.3) methodologies. On the other hand, N,N-dimethylglycine did not show a clear and distinguishable spectral signal after ultrafiltration, while in methanol precipitation, the presence of a baseline made its quantification difficult, and therefore, an increased error was expected. Similarly, 3-methyl-2-oxovalerate, isobutyrate and methionine neither showed a clear spectral signal in CPMG sequence, finding lipoprotein signals destabilizing the baseline. Citrate, an important tricarboxylic acid cycle intermediate, also displayed a high CV after g-SPE and ultrafiltration. This may be due to the long sample processing time, especially when applying ultrafiltration. Differential sample processing times can impact the enzymatic reactions occurring within the samples. In particular, plasma citrate, lactate and acetoacetate levels are known to decrease over time after sample collection, likely due to enzymatic degradation (Ghini et al., 2019). On the other hand, prolonged processing times can result in increased red blood cells lysis, thereby promoting the release of citrate synthase and subsequent conversion of glucose to citrate (Larsen et al., 2012). Brunius et al., also reported citrate as one of the features related to kinetic drift of NMR plasma metabolomics (Brunius et al., 2017). Citrate displayed a CV of 10.6% and 8.0% after the methanol precipitation and CPMG methodologies, respectively, which should be the techniques of choice for the quantification of this plasma metabolite. Despite g-SPE being a fast procedure, citrate was not generally detected. In line with these findings, Tiziani and co-workers found a lower concentration of citrate when a mix of methanol and chloroform was used for precipitating the plasmatic proteins (Tiziani et al., 2008), and Gowda et al. showed that citrate was also at higher concentration after ultrafiltration, compared with methanol precipitation (Nagana Gowda and Raftery, 2014).

**TABLE 1:** Compounds, absolute concentrations (mg/dL) and coefficient of variation (CV, %) in plasma pools (mean ± SD) using ^1^H-NMR spectroscopy. Metabolites are sorted alphabetically.

Metabolite	PR	SPE	UF	CPMG	*P* ^a^	FDR
2-Aminoisobutyrate^b^	1.950 ± 0.300 (15.4)	2.175 ± 0.150 (6.9)	2.400 ± 0.346 (14.4)	1.650 ± 0.173 (10.5)	0.005*	0.017*
2-Hydroxybutyrate	0.151 ± 0.040 (26.5)*	0.240 ± 0.019 (8.1)	0.082 ± 0.034 (41.9)*	0.090 ± 0.017 (19.3)	—	—
3-Hydroxybutyrate	1.071 ± 0.146 (13.7)	0.882 ± 0.064 (7.3)	1.086 ± 0.061 (5.6)	0.941 ± 0.148 (15.8)	0.072	0.143
3-Hydroxyisobutyrate	0.056 ± 0.005 (9.0)	0.061 ± 0.004 (5.9)	0.061 ± 0.006 (9.1)	0.049 ± 0.011 (22.2)	0.079	0.153
3-M-2-oxovalerate	0.178 ± 0.039 (22.1)*	0.181 ± 0.018 (9.8)	0.214 ± 0.020 (9.2)	0.058 ± 0.022 (37.6)*	<0.001*	<0.001*
Acetate	1.234 ± 0.524 (42.4)*	0.518 ± 0.353 (68.1)*	1.129 ± 0.835 (74.0)*	0.625 ± 0.325 (51.9)*	—	—
Acetoacetate	0.194 ± 0.045 (23.4)*	0.109 ± 0.017 (15.2)	0.361 ± 0.063 (17.5)	0.221 ± 0.014 (6.3)	<0.001*	<0.001*
Acetone	0.049 ± 0.005 (9.5)	0.066 ± 0.009 (13.1)	0.069 ± 0.017 (24.5)*	0.081 ± 0.004 (4.3)	0.009*	0.031*
Alanine	1.260 ± 0.074 (5.9)	1.335 ± 0.150 (11.2)	1.559 ± 0.029 (1.8)	1.107 ± 0.083 (7.5)	<0.001*	0.002*
Asparagine	0.590 ± 0.046 (7.9)	0.504 ± 0.094 (18.6)	0.486 ± 0.077 (15.8)	0.516 ± 0.100 (19.5)	0.320	0.411
Betaine	0.082 ± 0.002 (2.5)	0.092 ± 0.016 (17.6)	0.088 ± 0.014 (16.4)	0.064 ± 0.003 (4.8)	0.020*	0.055
Carnitine	0.303 ± 0.012 (3.9)	0.378 ± 0.076 (20.2)*	0.276 ± 0.072 (26.0)*	0.301 ± 0.032 (10.8)	—	—
Choline	0.064 ± 0.007 (11.3)	0.063 ± 0.008 (13.1)	0.063 ± 0.010 (15.8)	0.052 ± 0.008 (14.5)	0.195	0.294
Citrate	0.415 ± 0.050 (12.1)	0.024 ± 0.047 (200.0)*	0.948 ± 0.399 (42.0)*	0.564 ± 0.045 (8.0)	—	—
Creatine	0.214 ± 0.024 (11.1)	0.199 ± 0.011 (5.5)	0.224 ± 0.006 (2.6)	0.209 ± 0.024 (11.7)	0.316	0.409
Creatinine	0.487 ± 0.015 (3.1)	0.562 ± 0.058 (10.3)	0.566 ± 0.103 (18.2)	0.317 ± 0.036 (11.5)	<0.001*	<0.001*
Ethanol	0.318 ± 0.026 (8.2)	0.040 ± 0.005 (12.2)	0.229 ± 0.155 (67.7)*	0.046 ± 0.030 (64.6)*	—	—
Formate	1.279 ± 0.233 (18.2)	35.552 ± 26.926 (75.7)*	1.769 ± 2.186 (123.6)*	1.381 ± 0.000 (0)	—	—
Glucose	54.085 ± 2.088 (3.9)	60.719 ± 6.837 (11.3)	60.525 ± 2.416 (4)	59.999 ± 4.182 (7.0)	0.151	0.238
Glutamine	5.370 ± 0.222 (4.1)	4.745 ± 0.492 (10.4)	5.735 ± 0.362 (6.3)	4.368 ± 0.403 (9.2)	0.002*	0.008*
Glycerol	0.513 ± 0.080 (15.5)	0.695 ± 0.070 (10.1)	31.254 ± 10.223 (32.7)*	0.407 ± 0.028 (6.9)	—	—
Glycine	1.041 ± 0.148 (14.2)	0.828 ± 0.040 (4.9)	1.078 ± 0.104 (9.6)	1.194 ± 0.06 (5.0)	0.003*	0.013*
Histidine	0.750 ± 0.076 (10.2)	0.000 ± 0.000 (0)	0.855 ± 0.097 (11.3)	0.805 ± 0.127 (15.8)	0.001*	0.004*
Isobutyrate	0.050 ± 0.008 (16.4)	0.022 ± 0.006 (27.7)*	0.038 ± 0.012 (31.1)*	0.024 ± 0.006 (24.3)*	—	—
Isoleucine	0.444 ± 0.029 (6.6)	0.483 ± 0.041 (8.5)	0.491 ± 0.051 (10.5)	0.420 ± 0.025 (5.9)	0.072	0.143
Lactate	6.098 ± 0.521 (8.5)	3.598 ± 0.618 (17.2)	7.110 ± 0.804 (11.3)	4.728 ± 0.58 (12.3)	<0.001*	<0.001*
Leucine	0.905 ± 0.114 (12.6)	0.985 ± 0.051 (5.1)	0.988 ± 0.052 (5.3)	0.840 ± 0.043 (5.1)	0.035*	0.087
Lysine	1.319 ± 0.096 (7.3)	1.295 ± 0.050 (3.9)	1.482 ± 0.054 (3.7)	0.703 ± 0.025 (3.6)	<0.001*	<0.001*
Mannose	0.246 ± 0.053 (21.6)*	0.209 ± 0.040 (19.2)	0.247 ± 0.008 (3.3)	0.312 ± 0.056 (17.9)	0.031*	0.081
Methanol	8.530 ± 3.538 (41.5)*	0.369 ± 0.029 (7.9)	0.516 ± 0.180 (34.8)*	0.551 ± 0.443 (80.4)*	—	—
Methionine	0.224 ± 0.018 (7.8)	0.189 ± 0.033 (17.4)	0.205 ± 0.008 (4.1)	0.225 ± 0.050 (22.2)*	0.341	0.434
myo-Inositol	0.490 ± 0.066 (13.4)	0.414 ± 0.084 (20.3)*	0.286 ± 0.035 (12.2)	0.346 ± 0.044 (12.6)	0.003*	0.012*
N,N-Dimethylglycine	0.0082 ± 0.0056 (68.6)*	0.0051 ± 0.0028 (55.6)*	0.0046 ± 0.0040 (87.3)*	0.010 ± 0.001 (13.2)	—	—
O-Acetylcarnitine	0.129 ± 0.016 (12.3)	0.134 ± 0.025 (18.5)	0.126 ± 0.017 (13.6)	0.127 ± 0.015 (11.8)	0.928	0.949
Ornithine	0.360 ± 0.023 (6.3)	0.310 ± 0.022 (7.2)	0.364 ± 0.016 (4.3)	0.174 ± 0.007 (4.3)	<0.001*	<0.001*
Phenylalanine	0.465 ± 0.013 (2.7)	0.600 ± 0.054 (9)	0.531 ± 0.039 (7.3)	0.414 ± 0.072 (17.4)	0.001*	0.004*
Proline	0.888 ± 0.066 (7.4)	0.962 ± 0.040 (4.1)	0.973 ± 0.108 (11.1)	0.909 ± 0.053 (5.9)	0.312	0.408
Serine	0.758 ± 0.022 (2.9)	0.551 ± 0.024 (4.4)	0.662 ± 0.115 (17.4)	0.576 ± 0.111 (19.3)	0.016*	0.046*
Succinate	0.057 ± 0.006 (10.7)	0.051 ± 0.012 (22.7)*	0.056 ± 0.007 (12.7)	0.041 ± 0.005 (12.3)	0.052	0.114
Threonine	1.025 ± 0.045 (4.4)	1.037 ± 0.048 (4.7)	0.914 ± 0.038 (4.2)	0.817 ± 0.058 (7.1)	<0.001*	0.002*
*Tryptophan	0.551 ± 0.066 (12)	0.780 ± 0.011 (1.4)	0.000 ± 0.000 (0)	0.039 ± 0.046 (118.8)*	<0.001*	<0.001*
Tyrosine	0.559 ± 0.048 (8.6)	0.624 ± 0.040 (6.4)	0.621 ± 0.047 (7.6)	0.420 ± 0.064 (15.2)	<0.001*	0.002*
Valine	1.483 ± 0.132 (8.9)	1.358 ± 0.128 (9.4)	1.694 ± 0.072 (4.2)	1.337 ± 0.096 (7.2)	0.003*	0.012*

^*^Metabolites with a coefficient of variation > 20% in more than one group were removed from further analysis.

^a^ *p*-values from empirical distribution of permutation test with 5,000 iterations on multiclass Fisher scores.

^b^ Units for 2-aminoisobutyrate are in mM. 3-M-2-oxovalerate; 3-methyl-2-oxovalerate. FDR, Post-hoc based on false discovery rate; PR, methanol precipitation; SPE, glycerophospholipid-solid phase extraction; UF, ultrafiltration.

**FIGURE 2:**
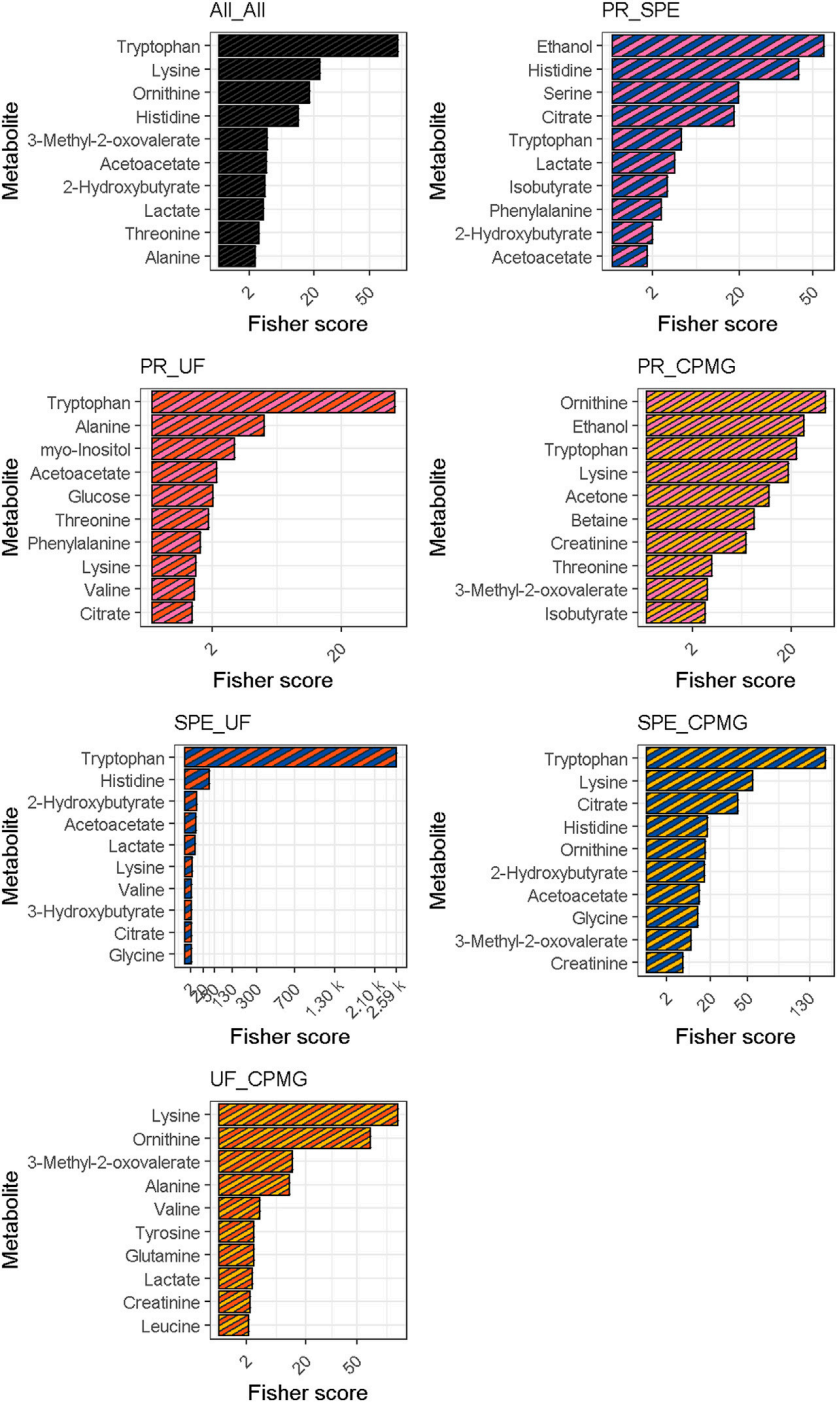
Fisher scores for multiclass and pairwise comparisons. Metabolites were sorted according to their median fisher criterion across the four comparisons. CPMG, Carr-Purcell-Meiboom-Gill; PR, methanol precipitation; SPE, glycerophospholipid-solid phase extraction; UF, ultrafiltration.

Fisher scores of measured metabolites are presented in Figure 2. The permutation test of multiclass Fisher scores showed that the concentrations of 18 metabolites were modified among the sample treatments with an FDR-corrected *p*-value < 0.05, shown in Table 1. Significant metabolites were then tested on a pairwise basis, shown in Table 2. The results showed that the efficiency of analyte extraction depended on the metabolite and the methodology utilized. Pairwise analysis denoted that concentrations of tryptophan, ornithine and acetoacetate were different among nearly all the procedures. While compared to CPMG, the techniques of SPE and ultrafiltration appeared to display the greatest differences, methanol precipitation and ultrafiltration seemed to exhibit more similar results.

**TABLE 2:** Pairwise Fisher scores comparisons between sample treatments.

Metabolite	PR:SPE	FDR	PR:UF	FDR	PR:CPMG	FDR	SPE:UF	FDR	SPE:CPMG	FDR	UF:CPMG	FDR
2-Aminoisobutyrate (uM)	0.251	0.353	0.102	0.180	0.161	0.249	0.271	0.370	<0.001*	0.003*	0.003*	0.012*
Acetoacetate	0.012*	0.037*	0.004*	0.014*	0.277	0.373	<0.001*	<0.001*	<0.001*	<0.001*	0.003*	0.012*
Alanine	0.399	0.496	<0.001*	0.003*	0.041*	0.096	0.039*	0.095	0.046*	0.106	<0.001*	<0.001*
Creatinine	0.063	0.131	0.181	0.275	<0.001*	0.002*	0.946	0.956	0.001*	0.005*	0.005*	0.018*
Glutamine	0.070	0.142	0.131	0.216	0.008*	0.026*	0.023*	0.062	0.271	0.370	0.002*	0.010*
Glycine	0.040*	0.095	0.693	0.786	0.097	0.176	0.013*	0.040*	<0.001*	0.002*	0.105	0.184
Histidine	<0.001*	0.002*	0.139	0.223	0.495	0.600	<0.001*	<0.001*	0.001*	0.004*	0.582	0.696
Lactate	0.001*	0.004*	0.084	0.159	0.022*	0.059	<0.001*	0.002*	0.041*	0.096	0.005*	0.018*
Lysine	0.674	0.773	0.020*	0.055	<0.001*	0.002*	0.001*	0.004*	<0.001*	0.002*	<0.001*	<0.001*
myo-Inositol	0.200	0.300	0.002*	0.011*	0.016*	0.045*	0.036*	0.088	0.195	0.294	0.078	0.152
Ornithine	0.012*	0.039*	0.764	0.849	<0.001*	0.002*	0.006*	0.020*	<0.001*	<0.001*	<0.001*	<0.001*
Phenylalanine	0.001*	0.005*	0.017*	0.048*	0.208	0.309	0.082	0.158	0.004*	0.017*	0.031*	0.080
Serine	0.001*	0.004*	0.137	0.223	0.024*	0.063	0.105	0.184	0.638	0.744	0.301	0.395
Threonine	0.724	0.814	0.014*	0.042*	0.001*	0.004*	0.012*	0.038*	0.002*	0.008*	0.042*	0.098
Tryptophan	0.009*	0.030*	0.001*	0.006*	0.001*	0.006*	<0.001*	<0.001*	<0.001*	0.002*	0.209	0.309
Tyrosine	0.081	0.156	0.112	0.191	0.013*	0.040*	0.929	0.949	<0.001*	0.003*	0.001*	0.006*
Valine	0.216	0.313	0.033*	0.083	0.119	0.199	0.008*	0.027*	0.800	0.875	0.003*	0.012*

*Statistically significant.

^a^ *p*-values from empirical distribution of permutation test with 5,000 iterations on pairwise Fisher scores. CPMG, Carr-Purcell Meiboom-Gill experiment; FDR, Post-hoc based on false discovery rate; PR, methanol precipitation; g-SPE, glycerophospholipid-solid phase extraction; UF, ultrafiltration.

Overall, branched-chain amino acid related valine, 3-methyl-2-oxovalerate and 2-aminoisobutyrate, aromatic amino acids such as phenylalanine, and tyrosine, and other amino acids such as alanine, glutamine, lysine, threonine and ornithine, along with creatinine, were found at lower concentrations in CPMG compared with the other sample treatments. On the other hand, acetone and glycine were found at higher concentration in untreated samples followed by CPMG experiments compared to the three pre-analytical treatments. One possible explanation for the lower concentrations in CPMG experiments is the lack of additional steps for concentrating and isolating metabolites with weak or overlapping signals (Weljie et al., 2006). Ultrafiltration, for example, employs a selective membrane to remove high molecular weight proteins and retain smaller metabolites. This process effectively concentrates the target analytes, resulting in higher concentrations. Similarly, SPE utilizes specific sorbents to extract metabolites, enabling their enrichment and subsequent elution for analysis (Tulipani et al., 2015). Methanol precipitation involves the addition of methanol to the plasma samples, causing protein precipitation and facilitating the recovery of the supernatant containing metabolites. These concentration steps can contribute to higher metabolite levels compared to CPMG experiments, where no such concentration steps are involved (Weljie et al., 2006). Another issue in the use of CPMG pulse sequences involves a series of refocusing pulses to counteract the signal decay caused by transverse relaxation (T2) processes. This approach can be affected by metabolite-macromolecule interactions, leading to reduced signal intensities and consequently lower observed concentrations. Metabolites in plasma samples have the potential to interact with macromolecules such as proteins, lipoproteins, and other biomolecules (Barrilero et al., 2017). It is worth noting that the binding of metabolites to macromolecules can vary depending on factors such as metabolite structure, pH, ionic strength, and the composition of the sample matrix (Kirwan et al., 2018). Different metabolites exhibit varying affinities for macromolecules, leading to differential effects on their signal intensities and concentrations in untreated samples using CPMG experiments and treated with ultrafiltration. This binding can occur through electrostatic interactions, hydrogen bonding, or hydrophobic interactions. As a result, the signals originating from bound metabolites decay more rapidly during the relaxation period compared to the signals from freely mobile metabolites (Bliziotis et al., 2020). The reduction of signal intensities for bound metabolites during the time relaxation period can result in underestimation of their concentrations in CPMG experiments (Nagana Gowda et al., 2018). Since the concentration determination is based on the signal intensities, the reduced signals of bound metabolites contribute to the overall lower concentrations observed in plasma samples prepared using CPMG compared to methods that involve additional processing steps like SPE or methanol precipitation (Nagana Gowda and Raftery, 2014). This was particularly observed for certain aromatic amino acids such as tryptophan (0.039 ± 0.046 mg/dL) and phenylalanine (0.414 ± 0.072 mg/dL) in CPMG experiments. Likewise, a total absence of tryptophan signals was found after ultrafiltration while in methanol precipitation, tryptophan concentration was 0.551 ± 0.066 mg/dL and even higher after g-SPE (0.780 ± 0.011 mg/dL). Since most of the plasma tryptophan is bound to albumin in the blood (McMenamy and Oncley, 1958), other metabolites bound to protein, such as creatinine (Varshney et al., 2011; Wallmeier et al., 2017), lactate (Bell et al., 1988; Chatham and Forder, 1999), tyrosine and histidine (Nicholson and Gartland, 1989), may require a protein degradation, such as methanol precipitation or SPE, to be correctly quantified in NMR metabolomics analysis. Nevertheless, in our results, the high levels of lactate in both ultrafiltration and methanol precipitation compared to those of g-SPE and CPMG are remarkable whereas histidine was not detected after g-SPE but was found to be at regular concentration in CPMG. Creatinine (0.317 ± 0.036 mg/dL) and tyrosine (0.420 ± 0.064 mg/dL) were found at lower concentrations uniquely in untreated samples processed using CPMG sequence. A study by Gowda and colleagues exhibited higher concentration of citrate after the ultrafiltration of 300 µL of serum compared to methanol precipitation. In contrast with the present study, the authors were able to quantify tryptophan after the ultrafiltration (0.474 ± 0.004 mg/dL). These results might be due to the free fraction of the metabolite in a larger amount of serum utilized. Nevertheless, the authors also reported that tryptophan concentration was much lower in ultrafiltration compared to methanol precipitation (1.291 ± 0.049 mg/dL) (Nagana Gowda and Raftery, 2014). Compared to ultrafiltration, the ketones acetoacetate and 3-hydroxybutyrate decreased after g-SPE, while levels of phenylalanine, tryptophan, and threonine were higher in ultrafiltration. Considering that ultrafiltration is the longest methodology, lipolytic action through microbial degradation could explain the lower levels of ketone bodies in ultrafiltration at the expense of increased lactate. In a study on sample stability, Bernini et al. reported increased lactate, while glucose decreased within 2 h at room temperature after blood collection (Bernini et al., 2011). However, despite the methanol precipitation may deactivate bacterial degradation rapidly, levels of lactate after ultrafiltration and methanol precipitation were similar. In this regard, Pinto and colleagues observed the occurrence of enzymatic lipolytic action without microbial growth (Pinto et al., 2014). In our study, all samples were collected and immediately stored at −80°C, and the only time discrepancy occurred during sample treatment.

Plasma treatments specifically alter the concentrations of certain metabolites. For instance, the concentration of methanol, formate and glycerol was influenced by the sample treatments, which have modified concentrations of these metabolites inherent to the used procedures (Wishart, 2008; Ghini et al., 2019). A high concentration of methanol is related to a residue of the methanol used for the protein precipitation instead of a higher extraction of the endogenous metabolome. Although the mix with chloroform should improve the extraction of lipoproteins and lipids, the final ratio of methanol-plasma was set to 2:1 (v/v) (Nagana Gowda et al., 2015). Protein precipitation by using a 1:1 methanol-to-sample ratio retains a high level of residual proteins that complicates the identification/quantification (Snytnikova et al., 2019). Higher methanol-sample ratios may increase the spectral quality but also evaporation time and solvent residue, affecting sample stability (Crook and Powers, 2020). Likewise, a prominent singlet at δ8.49 corresponding to formate was present in the aromatic region of the spectra and it is related to a residue of the formate used during g-SPE treatment (Tulipani et al., 2013; González-Domínguez et al., 2020). Glycerol concentration was very elevated after ultrafiltration due to insufficient washing of filters (Ghini et al., 2019). These metabolites were then excluded from further analyses.

In the present study, the use of methanol precipitation retained ethanol and serine concentrations more effectively compared to g-SPE, while myo-inositol extraction was higher in the methanol precipitation and g-SPE groups. Other authors found a better recovery of most metabolites when g-SPE was compared to precipitation with methanol or ultrafiltration (Tulipani et al., 2013). The authors showed that the combination of solvent extraction and SPE-mediated removal of phospholipids, prior analysis by MS, was the most suitable sample preparation for detecting subtle quantitative changes for the majority of the remaining metabolites. The extraction of polar compounds such as acetylcholine, acetyl-l-carnitine, leucine, isoleucine, and phenylalanine was higher compared to ultrafiltration and solvent precipitation. With the exception of phenylalanine, none of these metabolites were statistically different across the methodologies used in the present study. Further optimization of the procedure may enhance the global extraction efficiency of this sample treatment. Interestingly, ethanol levels after ultrafiltration and CPMG were extremely unstable, and levels of this metabolite should be interpreted with caution when using these procedures. Many NMR-based metabolomics studies have used ultrafiltration due to its efficiency for protein removal with optimal metabolite extraction (Bathe et al., 2011; Psychogios et al., 2011; Farshidfar et al., 2012; Nagana Gowda and Raftery, 2014). Tiziani and colleagues reported that relative to the protein precipitation methods, ultrafiltration performs better in retaining metabolite concentrations (Tiziani et al., 2008). Nevertheless, this statement was discussed in other studies (Nagana Gowda et al., 2015). In the present study, several considerations have been encountered. After ultrafiltration, glycerol concentration was around 1.5 orders of magnitude higher than in methanol precipitation and g-SPE, despite that centrifugal filters were washed as published elsewhere (Nagana Gowda and Raftery, 2014). Further filter washing would critically increase sample handling and time preparation, and this may affect the stability of plasma/serum metabolites (Brunius et al., 2017).

In the present NMR metabolomics study, four methodologies for removing macromolecules in plasma samples, CPMG, ultrafiltration, methanol precipitation, and adapted g-SPE were evaluated. The results indicate that this step may be critical for the reliable quantitation of many metabolites, while the claimed reproducibility of NMR is also investigated. There are improvements in quantification performance between treatments that are specific to certain metabolites, finding differences that are metabolite-dependent. Each methodology also presents several considerations. For instance, the cost, the need to wash centrifugal filters several times critically increases the time procedure, and the loss of signal of protein-associated metabolites in plasma/serum samples are some of the limitations of ultrafiltration. On the other hand, in light of the present results, untreated plasma samples followed by CPMG experiments offer an attractive alternative in terms of processing time, cost and reliability of results, provided that limitations in the replicability of some metabolites and underestimation in the concentrations of several metabolites are considered. To mitigate the impact of metabolite-macromolecule binding, additional sample processing steps such as ultrafiltration, SPE, or methanol precipitation can be employed. These techniques aim to separate metabolites from macromolecules, thereby minimizing their interactions and allowing for more accurate quantification of metabolite concentrations. Although methanol precipitation is a popular sample treatment in plasma/serum samples, the extraction efficiency of methanol precipitation can depend on the solvent-to-sample ratio, and in our case, we have observed a baseline that made quantification difficult, whereas g-SPE provides spectra without macromolecule residues, and in terms of repeatability, it was the technique of choice. Yet, this treatment is also costly and presented various metabolites with higher concentrations while underestimating others. To conclude, this work highlights that the different methodologies affect extraction performance in a metabolite-dependent manner in quantitative metabolomics, and this allows the selection of the most appropriate sample treatment for each case.

## Data Availability

The original contributions presented in the study are included in the article/Supplementary Material, further inquiries can be directed to the corresponding authors.
