# Supplementary material for: Quantitative plasma profiling by 1H NMR-based metabolomics: impact of sample treatment
Source: Front Mol Biosci. 2023 Jun 2;10:1125582. doi: 10.3389/fmolb.2023.1125582 (PMC10273206; doi:10.3389/fmolb.2023.1125582)

Quantitative plasma profiling by ^{1}H NMR-based metabolomics: impact of sample treatment

Francisco Madrid-Gambin[1,2,*], Sergio Oller[2,3], Santiago Marco[2,3], Óscar J. Pozo[1], Cristina Andres-Lacueva[4-7], Rafael Llorach[4-7,*]

[1]Applied Metabolomics Research Group, IMIM - Institut Hospital del Mar d'Investigacions Mèdiques, 08003 Barcelona, Spain.

[2]Signal and Information Processing for Sensing Systems, Institute for Bioengineering of Catalonia (IBEC), The Barcelona Institute of Science and Technology, 08028 Barcelona, Spain.

[3]Department of Electronics and Biomedical Engineering, Faculty of Physics, University of Barcelona, 08028 Barcelona, Spain.

[4]Biomarkers and Nutrimetabolomics Laboratory, Department of Nutrition, Food Science and Gastronomy, Faculty of Pharmacy and Food Sciences, Campus Torribera, University of Barcelona, 08921 Sant Coloma de Gramanet, Spain.

[5]Food Innovation Network (XIA), Santa Coloma de Gramanet, Spain

[6]Institut de Recerca en Nutrició i Seguretat Alimentària (INSA-UB), Santa Coloma de Gramanet, Spain.

[7]Centro de Investigación Biomédica en Red de Fragilidad y Envejecimiento Saludable (CIBERFES), Instituto de Salud Carlos III, Madrid 28029, Spain.

*** Correspondence:**
Francisco Madrid-Gambin. fmadrid@imim.es

Rafael Llorach. rafallorach@ub.edu

Keywords: metabolomics, nuclear magnetic resonance, plasma, pre-analytical treatment, quantitative analysis, quantification.

Supplemental Figure 1. Concentration (mg/dL) of detected metabolites (page 1/2).

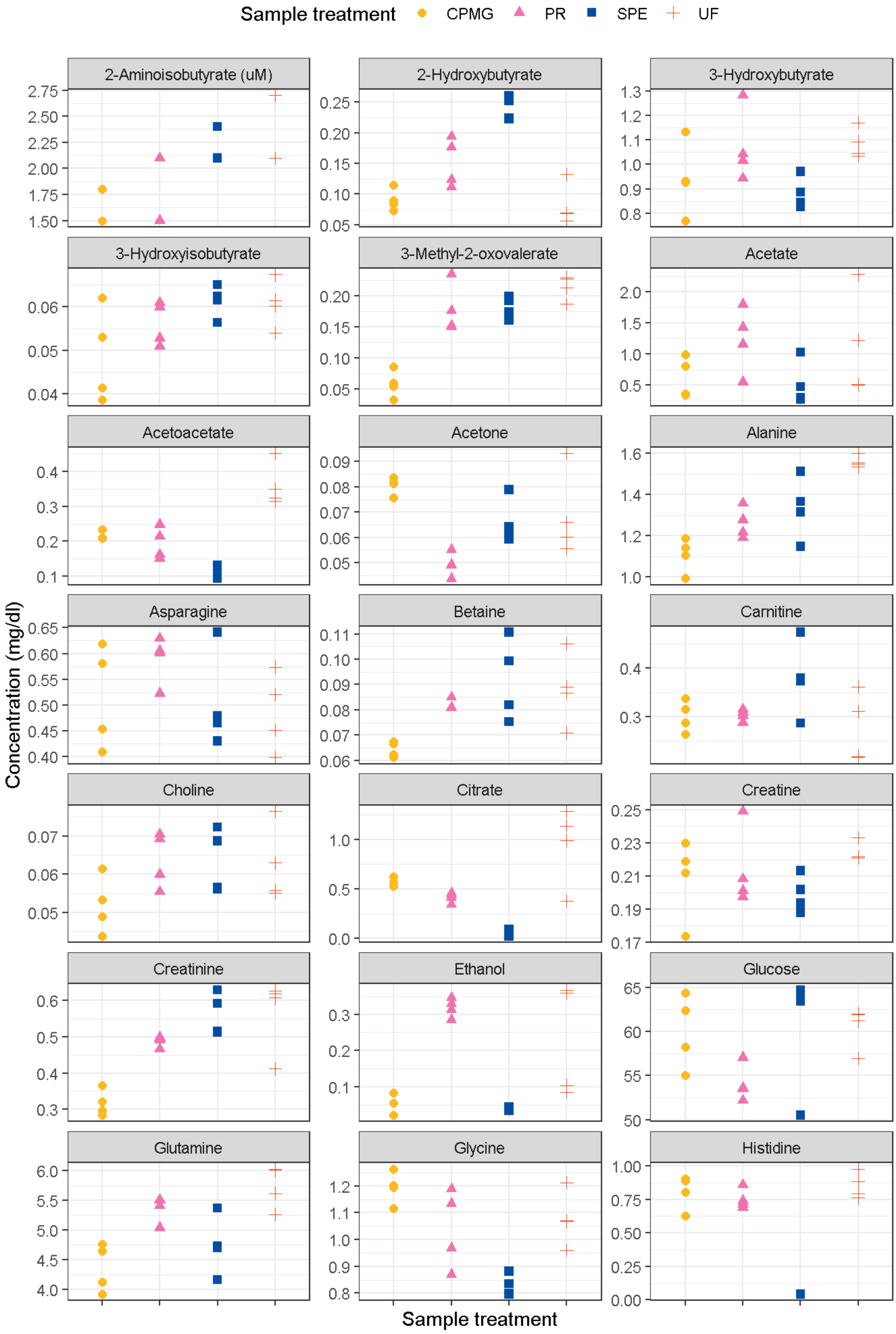

Supplemental Figure 1 (Page 2/2).

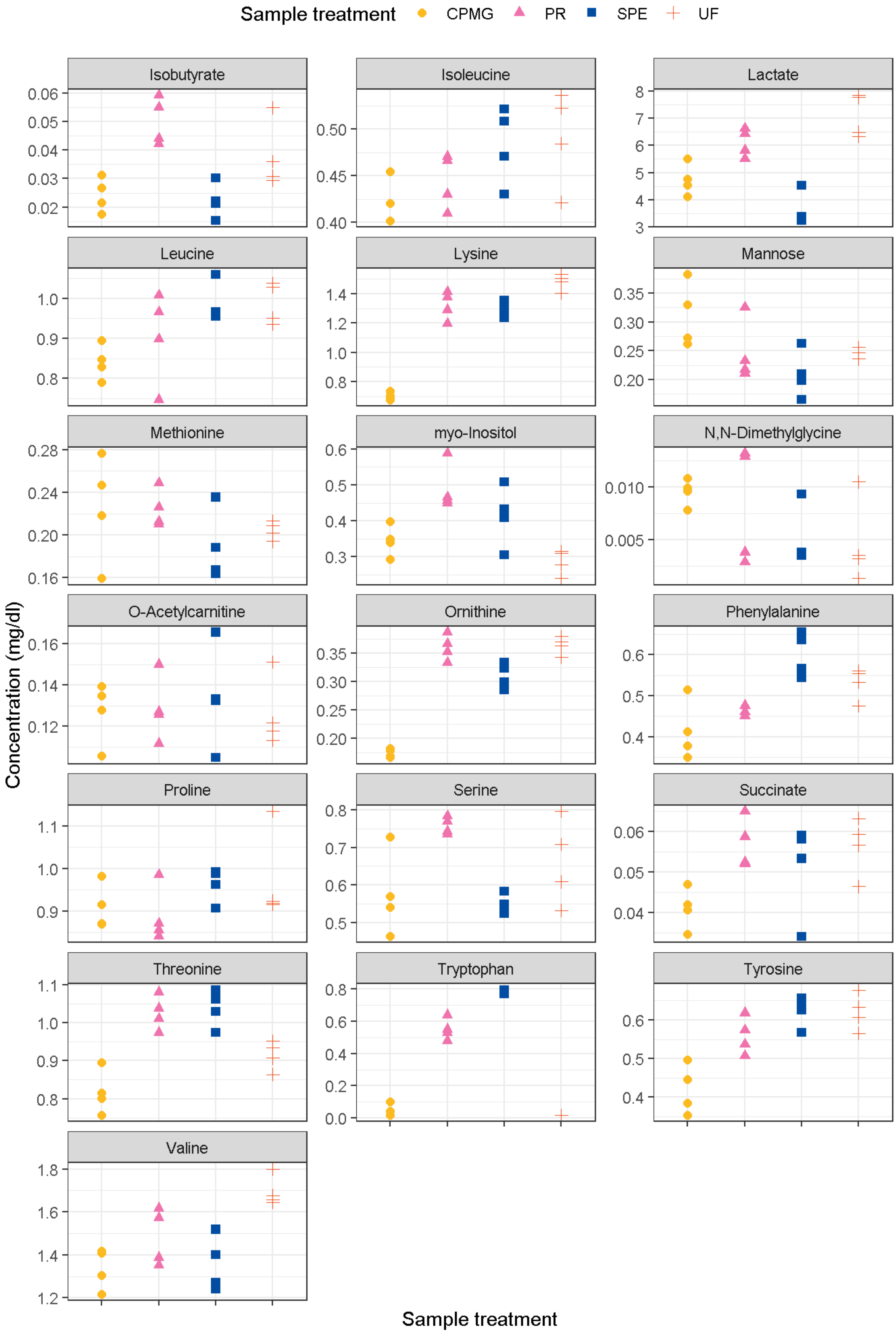

Supplemental Figure 2. Supplemental Figure 1. Signal of the reference standard sodium trimethylsilyl propionate-2,2,3,3-d_4 (TSP) of treated plasma samples acquired with Nuclear Overhauser Effect Spectroscopy (NOESY, in black) and an untreated plasma sample the Carr-Purcell-Meiboom-Gill (CPMG, in red) pulse sequences.

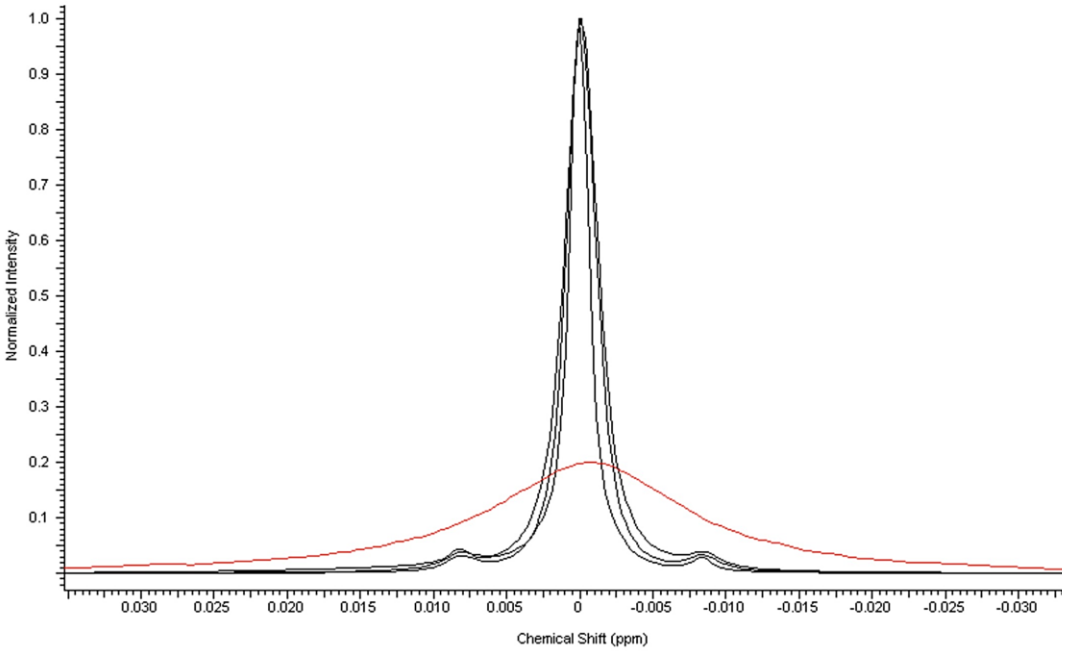

Supplemental Figure 3. Comparison of fisher scores for metabolites across all method

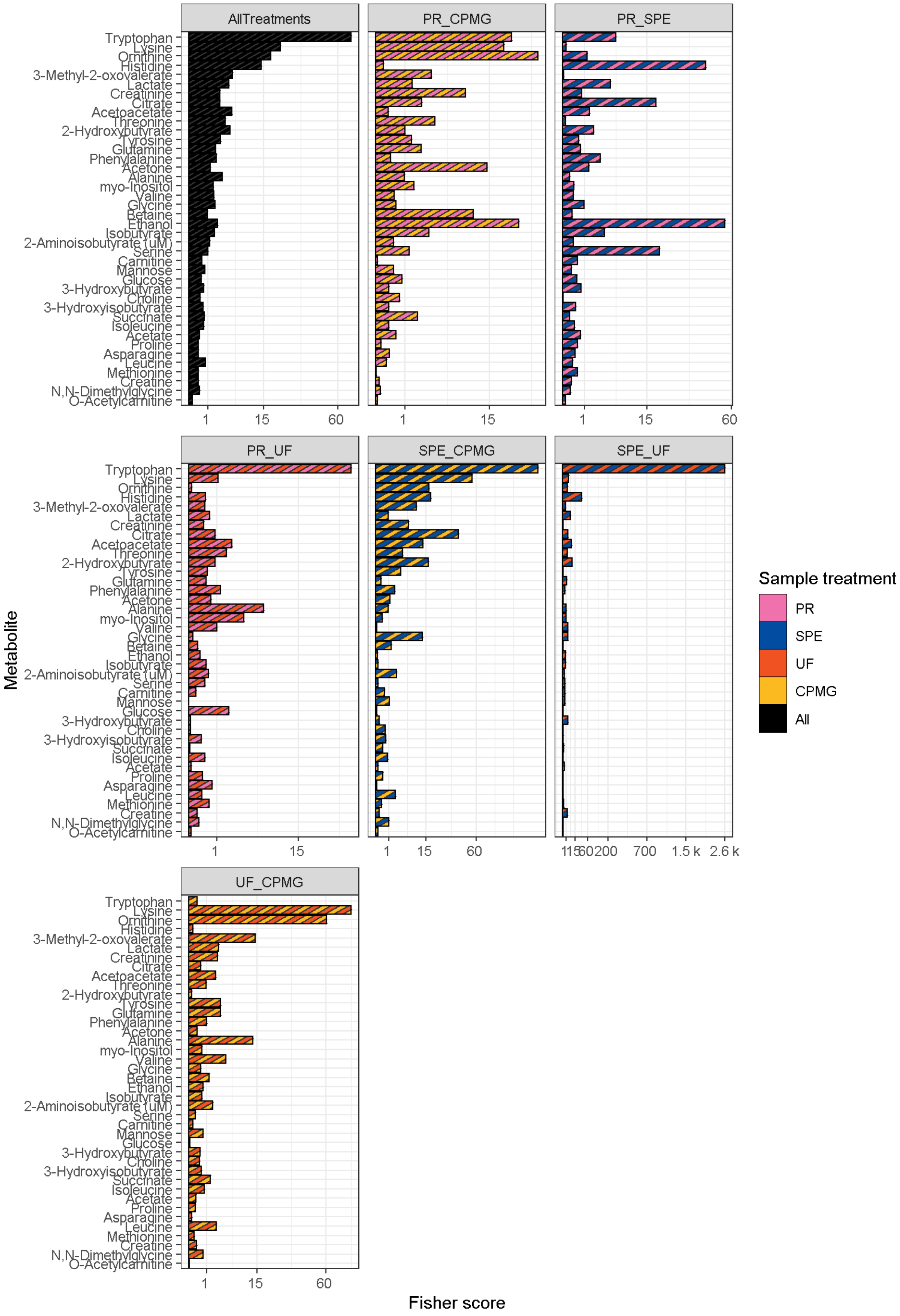

Supplement: Supplementary file 1 [file DataSheet1.pdf]
